# Anatomical Adaptation of Fibula and its Mechanism of Proximal Partial Fibulectomy Associated with Medial Compartment Knee Osteoarthritis

**DOI:** 10.1111/os.12437

**Published:** 2019-04-06

**Authors:** Juan Wang, Hong‐zhi Lv, Wei Chen, Meng‐ke Fan, Ming Li, Ying‐ze Zhang

**Affiliations:** ^1^ Editorial Department The Third Hospital of Hebei Medical University Shijiazhuang China

**Keywords:** Knee osteoarthritis, medial compartment, proximal partial fibulectomy

## Abstract

**Objectives:**

To reveal the anatomical adaptation of the fibula and its relations to age and settlement of the medial tibial plateau, and to explore the mechanism of proximal partial fibulectomy in treating medial compartment knee osteoarthritis (OA).

**Methods:**

A retrospective study was performed in the Third Hospital of Hebei Medical University. Weight‐bearing full‐leg anteroposterior (AP) radiographs of 280 adults (560 knees) obtained from 1 January 2018 to 31 October 2018 were enrolled according to our inclusion and exclusion criteria, including 157 men and 123 women, with an average age of 50.3 ± 14.8 years (range, 19–80 years). Radiographic severity of knee OA was assessed using Kellgren and Lawrence (K–L) grading. The settlement of the medial tibial plateau was evaluated using the medial proximal tibial angle (MPTA). Curvatures of the tibia and the fibula were measured as proximal tibial curvature (PTC), distal tibial curvature (DTC), proximal fibular curvature (PFC), and distal fibular curvature (DFC). Two orthopaedic surgeons performed all the radiological measurements for 30 randomly selected patients, and repeated the measurements 1 week later. Based on the satisfactory intra‐observer and inter‐observer reliabilities (ICC > 0.9), each parameter was analyzed in this study. Multivariable linear regression models were used to examine relations between radiological measurements and age.

**Results:**

The mean MPTA, PTC, DTC, PFC, and DFC were 85.4° ± 2.8°, 176.2° ± 1.9°, 176.8° ± 1.8°, 176.8° ± 1.9°, and 177.0° ± 2.0°, respectively. Ninety‐three knees of K–L grade I were categorized as non‐knee OA, and 467 knees of K–L grades II–IV were categorized as knee OA. The MPTA, PTC, and PFC of the knee OA group were significantly smaller than those of non‐knee OA group (*P* < 0.05). The K–L grade of knee OA significantly increased with age (*χ* ^*2*^ = 182.169, *P <* 0.01). The multivariate linear regression analysis indicated that the MPTA and fibular curvatures were negatively correlated with age (the regression equation is age = 561.165–0.945 MPTA‐0.937 PFC‐0.959 DFC, *P* < 0.05), and the MPTA was negatively correlated with PFC (the regression equation is MPTA = 7.827 + 0.099 DFC, *P* < 0.05).

**Conclusions:**

The proximal curve of the fibula increased in patients with medial compartment knee OA, and this change was positively correlated with age and settlement of the medial tibial plateau. This anatomical adaptation of the fibula was associated with greater fibular axial load and the pulling from the peroneus longus. The proximal partial fibulectomy procedure effected a receptive foot pronation to reduce KAM and rebalance the biceps‐proximal fibula–peroneus longus complex, consequently achieving medial compartment unloading.

## Introduction

Medial compartment osteoarthritis (OA) is the most common subtype of end‐stage knee OA in China, which disables middle‐aged and elderly sufferers and necessitates total knee arthroplasy (TKA) as the definitive treatment^1^, ^2^. Before progressing to panarthritis, open wedge high tibial osteotomy (HTO) or medial unicompartmental arthroplasty (UKA) may be advised to address the medial compartment involvement in knee OA, with the aim to avoid or postpone TKA. These major surgeries all have very strict indications, are costly, and are relatively high risk. Therefore, many patients with knee OA are still undertreated due to medical or/and non‐medical barriers, including, in our experience, contraindications for the abovementioned procedures, financial difficulties, and fear of major surgery. Based on these circumstances, establishing alternative solutions to alleviate symptoms and prevent progression of knee OA is urgently needed.

**Figure 1:**
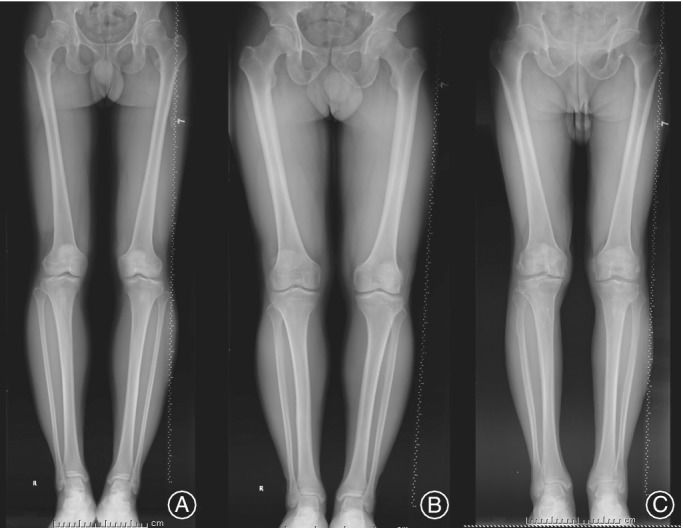
Three representative lower extremity weight‐bearing full‐length images showing fibular curvature change with age. (A) This patient was a 27‐year‐old male, with proximal fibular curvature of 176.9 (left) and 177.4 (right). (B) This patient was a 40‐year‐old man, with PFC of 174.6 (left) and 175.3 (right). (C) This patient was a 78‐year‐old man, with PFC of 172.9 (left) and 170.1 (right).

Partial fibulectomy is a procedure commonly used to harvest bone graft or to treat nonunion of tibial fractures or fibular fractures^3^. One previous cadaveric study revealed that partial fibulectomy at 12 cm above the lateral malleolus resulted in decreased pressure in the medial compartment and increased pressure in the lateral compartment of the knee joint^4^. However, this study was irrelevant to knee OA. Proximal fibular osteotomy has been used in treating knee OA as a supplementary procedure to the closed wedge HTO. As an innovative clinical application of this procedure, partial fibulectomy at 6–10 cm below the fibular head without concomitant HTO, referred to as proximal fibular osteotomy or proximal partial fibulectomy in the literature, has been performed to treat medial compartment knee OA in our institute since the 1990s. Our clinical and radiological follow‐up showed decreased mean femorotibial angle (FTA), lateral joint space, and mean visual analog scale (VAS) score, as well as improved American Knee Society Score (KSS) and Hospital for Special Surgery (HSS) knee score, indicating that proximal fibular osteotomy can significantly improve both the radiographic appearance and the function of the affected knee joint, and also achieve long‐term pain relief (Fig. 1)^5^, ^6^. Recent published studies have consistently reported improvements in pain and function, as well as re‐adjustment of knee biomechanics and kinematics after proximal partial fibulectomy^7^, ^8^, ^9^, ^10^. The application of this procedure in treating medial compartment knee OA was rationalized by the theory of non‐uniform settlement of the tibial plateau, which contains two essential initiatives that work synergistically to exacerbate the varus deformity of the tibia: osteoporosis of the proximal tibia and lateral support provided by the fibula–soft tissue complex^11^. However, how the fibula is involved anatomically, biomechanically, and kinematically in the pathological process of medial compartment knee OA is yet to be clarified.

The fibula is not directly involved in the constitution of the knee joint and its function in weight‐bearing is minor. For this reason, this bony structure has been ignored in research regarding knee OA. The fibula is S‐shaped, with its proximal half curving towards posterolateral and the distal half towards posteromedial. It was shown that the when axial load was applied either through the distal femur or direct to the tibial plateau, the fibula tended to bend posterolaterally, which was constrained by the interosseous membrane, and the fibular strains were not reduced to zero following sectioning of the interosseous membrane^12^. Goh *et al.* reported that the load distribution to the fibula averaged 7.12% of the total force transmitted through the tibia and fibula with the ankle at neutral position, and maximum loads occurred at full dorsiflexion and eversion^13^. Levinger *et al.* also reported that knee OA patients exhibited a more pronated foot posture (with rearfoot eversion) compared to controls^14^. Accordingly, the compensational eversion rearfoot posture in patients with varus malalignment of the knee joint may increase the axial load transmitted through the fibula, thus creating increased posterolateral bending force acting on the fibula shaft. This directed our attention to the curvature of the fibula in patients with varus type of knee OA in our clinical practice. The senior author of this study noticed that in many elderly patients with obvious tibia vara observed on anterior–posterior X‐ray films, the curvature of the proximal half of the fibula was increased compared to young adults. Based on the published literature and our clinical observations, we hypothesized that anatomical adaptation of the fibula occurs when a patient with medial compartment knee OA is aging and the non‐uniform settlement of the tibia is progressing; the anatomical change of the fibula further demonstrates the kinematic effect of the fibula and proximal partial fibulectomy on medial compartment knee OA.

The purposes of the current study were to: (i) reveal the anatomical change of the fibular curvatures *via* retrospective review of the radiograph database in our institute; (ii) reveal the relations of the fibular curvatures to age and settlement of the medial tibial plateau; and (iii) explore the effective mechanism of proximal partial fibulectomy in treating medial compartment knee OA, thus further rationalizing this innovative application of the procedure.

## Patients and Methods

This study was approved by the review board of our institute. Informed consent was obtained from all individual participants who was enrolled in this study. Weight‐bearing full‐leg X‐ray images acquired in our hospital between 1 January 2018 and 31 October 2018 were reviewed through the PACS system. Full‐leg standing digital AP radiography was performed for 395 patients during this period. The inclusion criteria were as follows: (i) older than 18 years; and (ii) standard lower extremity weight‐bearing full‐length film. The exclusion criteria of this study were: (i) genu valgum; (ii) pagoda tibia; (iii) severe extension deficit of the knee; (iv) history of trauma or surgery of either lower extremity; (v) skeletal dysplasia or osteopathy involving lower extremities; and (vi) inflammatory arthritis and other diseases involving knee joints. Based on these criteria, 115 patients were excluded, and 280 patients (560 knees) were included and underwent radiological measurement. All images were evaluated independently by three registrars specializing in orthopaedics on two occasions separated by a 1‐week interval.

### *Radiological Measurements*

In this study, radiographic severity of knee OA was assessed using Kellgren and Lawrence (K–L) grading. Radiographic knee OA was defined as K–L grades II–IV^15^. Grade 0 indicated a definite absence of X‐ray changes in osteoarthrosis: grade I indicated suspicious narrowing of joint clearance and possible osteophytes; grade II indicated obvious osteophytes and slight narrowing of the joint space; grade III indicated moderate osteophytes, narrowing of joint space, mild sclerosis of subchondrobone and small range; and grade IV indicated a large number of osteophytes forming, which can spread to the chondroid surface, with the joint gap becoming narrower, extremely obvious hardening changes, joint hypertrophy, and obvious deformity.

**Figure 2:**
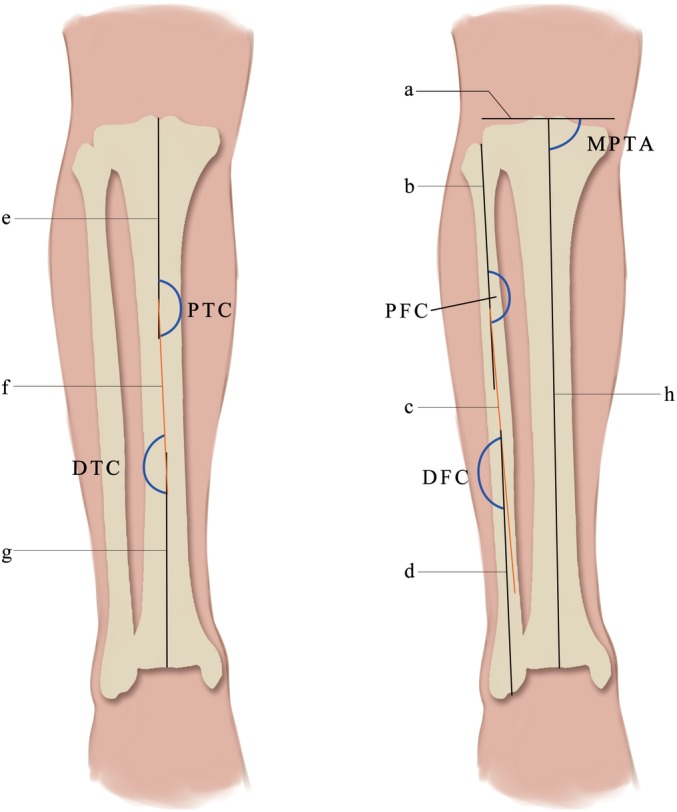
Measurement diagram of medial proximal tibial angle (MPTA), proximal tibial curvature (PTC), distal tibial curvature (DTC), proximal fibular curvature (PFC), distal fibular curvature (DFC) on weight‐bearing full‐leg digital anteroposterior (AP) radiographs. The MPTA was defined as the medial angle between the tangent to the tibial plateau line (a) and the mechanical axis of the tibia line (h). The tibia and the fibula were divided into different segments by turning points of the medullary cavity central line. The PTC was defined as the obtuse angle between the proximal medullary cavity central line (e) and the middle medullary cavity central line (f) of the tibia, and DTC was defined as the obtuse angle formed by the middle medullary cavity central line (f) and the distal medullary cavity central line (g) of the tibia. The PFC and DFC were defined as the obtuse angles formed by the proximal medullary cavity central line (b) and the middle medullary cavity central line (c) of the fibula, and the distal medullary cavity central line (d) and the middle medullary cavity central line (c) of the fibula, respectively.

The radiological measurements included: (i) medial proximal tibial angle (MPTA), indicating the degree of medial plateau settlement; (ii) proximal tibial curvature (PTC) and distal tibial curvature (DTC), indicating anatomical changes of the tibia; and (iii) proximal fibular curvature (PFC) and distal fibular curvature (DFC), indicating anatomical changes of the fibula. The method we designed to measure angles is shown in Fig. 1. The MPTA was defined as the medial angle between the tangent to the tibial plateau line and the mechanical axis of the tibia line. The tibia and the fibula were divided into different segments by turning points of the medullary cavity central line. The PTC was defined as the obtuse angle between the proximal medullary cavity central line and the middle medullary cavity central line of the tibia, and DTC was defined as the obtuse angle formed by the middle medullary cavity central line and the distal medullary cavity central line of the tibia. The PFC and DFC were defined as the obtuse angles formed by the proximal medullary cavity central line and the middle medullary cavity central line of the fibula, and the distal medullary cavity central line and the middle medullary cavity central line of the fibula, respectively (Fig. 2).

A computerized goniometer of the Synapase System (Fujifilm Medical Systems, Stamford, USA) was used for measurement. Two orthopaedic surgeons performed all the radiological measurements by two investigators for 30 randomly selected patients, and repeated them 1 week later. Intra‐observer and inter‐observer reliabilities were estimated by intraclass correlation coefficients (ICC), with an ICC greater than 0.8 considered as having excellent reliability. Based on the satisfactory intra‐observer and inter‐observer reliabilities (ICC > 0.9) for each measurement, radiological measurements from one same investigator were analyzed in this study.

### *Statistical Analyses*

Statistical analyses were carried out with SPSS 21.0 (IBM, Armonk, New York) software for Windows. The normality of continuous variables was assessed using the Kolmogorov–Smirnov test. Continuous variables in line with normal distribution were described as the mean ± SD and categorical variables were described as frequencies. Independent *t*‐test analysis of variance was performed to compare the MPTA, PTC, DTC, PFC, and DFC between non‐knee OA and knee OA groups. Kruskal–Wallis H analysis of variance was performed to evaluate the significance of differences between various age groups with regard to MPTA, PTC, DTC, PFC, and DFC. The linear trend K–L grade by age was analyzed using the linear by linear χ^2^‐test. Multiple linear regression analyses were performed to detect the relationship between MPTA angle, age, and curvatures of the tibia and the fibula. The level of significance was defined as *P* < 0.05.

## Results

### *Demographic Information and Radiological Measurement*

Two hundred and eighty patients (560 knees) were enrolled in the study, including 157 men and 123 women, with an average age of 50.3 ± 14.8 years (range, 19–80 years). The mean MPTA was 85.4° ± 2.8° (range, 72.7° to 89.9°). The mean PTC and DTC were 176.2° ± 1.9° (range, 166.2°–180°) and 176.8° ± 1.8° (range, 171.1°–180°), respectively. The mean PFC and DFC were 176.8° ± 1.9° (range, 169.9°–180°) and 177.0° ± 2.0° (range, 170°–180°), respectively. Ninety‐three knees were graded as K–L grade I and categorized into the non‐knee OA group. The remaining knees, including 230 knees of K–L grade II, 189 of KL grade III, and 48 of K–L grade IV, were categorized into the knee OA group.

### *Comparison of Radiological Measurements between Knee Osteoarthritis and Non‐knee Osteoarthritis Groups*

**Figure 3:**
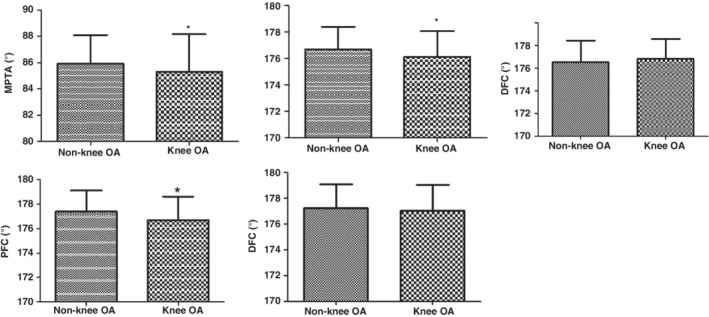
Differences of radiological measurements between knee osteoarthritis (OA) and non‐knee OA groups. *Compared to non‐knee osteoarthritis (OA) group; *P* < 0.01.

In the non‐knee OA group, the mean MPTA, PTC, DTC, PFC, and DFC were 85.89° ± 2.20°, 176.68° ± 1.70°, 176.56° ± 1.86°, 177.39° ± 1.70°, and 177.23° ± 1.88°, respectively. In the knee OA, the mean MPTA, PTC, DTC, PFC, and DFC were 85.30° ± 2.85°, 176.11° ± 1.93°, 176.81° ± 1.77°, 176.68° ± 1.92°, and 177.01° ± 2.01°, respectively. The MPTA, PTC, and PFC were significantly smaller in the knee OA group compared to the non‐knee OA group (*P* < 0.05, Fig. 3, Table 1), while the DTC and DFC showed no significant difference between groups.

**Table 1:** Differences of radiological measurements between knee osteoarthritis (OA) and non‐knee OA groups (°, $\bar{x}$ ± s)

	*n*	MPTA	PTC	DTC	PFC	DFC
KOA	93	85.9±2.2	176.7 ± 1.7	176.6 ± 1.9	177.4 ± 1.7	177.2±1.9
non‐KOA	468	85.3±2.9	176.1±1.9	176.8±1.8	176.7±1.9	177.0±2.0
*t*‐value		2.238	2.820	−1.257	3.540	1.017
*P*‐value		0.027	0.005	0.211	0.001	0.311

MPTA, medial proximal tibial angle; PTC, proximal tibial curvature; DTC, distal tibial curvature; PFC, proximal fibular curvature; DFC, distal fibular curvature.

### *Variation Trend of Radiological Measurements by Age*

**Figure 4:**
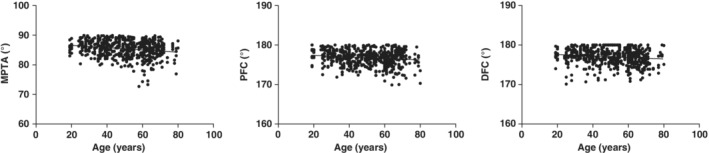
Scatterplot and linear correlation between age and medial proximal tibial angle (MPTA), proximal fibular curvature (PFC) and distal fibular curvature (DFC).

The results revealed significant differences in MPTA, PTC, DTC, PFC, and DFC among different age groups. The MPTA (86.4° ± 2.0°), PTC (176.9° ± 1.7°), PFC (177.7° ± 1.7°), and DFC (177.2° ± 2.1°) of 19–29‐year‐old patients were all significantly higher than those of ≥70‐year‐old patients (84.7° ± 2.7°, 176.7° ± 1.5°, 176.6° ± 2.3°, 176.4° ± 1.9°, *P <* 0.01, Table 2). The DTC of 19–29‐year old patients (176.7° ± 1.8°) were all significantly lower than those of ≥70‐year‐old patients (177.3° ± 1.4°, *P <* 0.01, Table 2). Multivariate linear regression analysis showed that the MPTA and fibular curvatures were negatively correlated with age, indicating greater settlement of the medial tibial plateau and fibular curves in older patients (age = 561.165–0.945 MPTA‐ 0.937 PFC‐ 0.959 DFC, *P <* 0.01, Table 3). The K–L grade of knee OA in this study showed a significant tendency to increase with age; the proportion of patients with knee OA among the 19–49 year olds (66.4%) was significantly less than that among the ≥50 year olds (97.4%, *χ* ^*2*^ = 182.169, *P <* 0.01, Table 4, Fig. 4).

**Table 2:** Radiological measurements in different age groups (°, $\bar{x}$ ± s)

Age (years)	*n*	MPTA	PTC	DTC	PFC	DFC
19–29	61	86.4±2.0	176.9 ± 1.7	176.7 ± 1.8	177.7 ± 1.7	177.2 ± 2.1
30–39	91	85.9±2.4*	176.5 ± 1.7*	176.7 ± 1.7	177.0 ± 1.7*	177.5 ± 1.7
40–49	101	86.0 ± 2.6*	176.2 ± 2.1	177.5 ± 1.6	176.6 ± 1.8*	177.1 ± 2.0
50–59	99	85.0 ± 2.9*	175.4 ± 2.2	176.6 ± 1.8*	176.9 ± 1.7*	177.2 ± 1.9
60‐69	164	84.8 ± 3.1*	176.1 ± 1.7	176.4 ± 1.9*	176.5 ± 2.1*	176.7 ± 2.1
≥70	44	84.7 ± 2.7*	176.7 ± 1.5*	177.3 ± 1.4*	176.6 ± 2.3*	176.4 ± 1.9*
*χ* ^*2*^‐value		22.907	26.451	28.458	20.745	17.895
*P*‐value		<0.001	<0.001	<0.001	0.01	0.003

* Compared to 19–29 group, *P* < 0.01. MPTA, medial proximal tibial angle; PTC, proximal tibial curvature; DTC, distal tibial curvature; PFC, proximal fibular curvature; DFC, distal fibular curvature.

**Table 3:** Correlations between radiological measurements and age by multivariate linear regression analysis

Constant	Partial regression coefficient	Standardized coefficients β	*t*‐value	*P*‐value	95%CI		
	561.165	‐	5.267039	0.000	351.888	‐	770.442
MPTA	−0.945	−0.177	−4.26003	0.000	−1.381	‐	−0.509
PTC	−0.414	−0.053	−1.2935	0.196	−1.043	‐	0.215
DTC	−0.123	−0.015	−0.36126	0.718	−0.794	‐	0.547
PFC	−0.937	−0.121	−2.91287	0.004	−1.569	‐	−0.305
DFC	−0.959	−0.129	−3.14221	0.002	−1.558	‐	−0.359

MPTA, medial proximal tibial angle; PTC, proximal tibial curvature; DTC, distal tibial curvature; PFC, proximal fibular curvature; DFC, distal fibular curvature; CI, confidence interval.

**Table 4:** Kellgren and Lawrence grading of knee OA in different age groups (knees)

Age (years)	0	1	2	3	4
19–29	0	24	33	4	0
30–39	0	41	43	7	0
40–49	0	20	51	23	7
50–59	0	8	47	33	11
60–69	0	0	48	95	21
≥70	0	0	8	27	9
*χ* ^*2*^‐value	182.169				
*P*‐value	<0.01				

OA, osteoarthritis

### *Correlation between medial proximal tibial angle and Curvatures of Tibia and Fibula*

**Figure 5:**
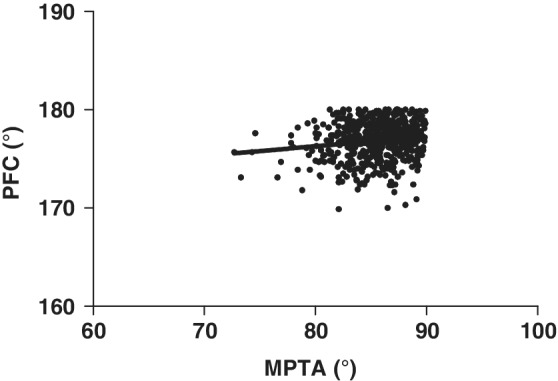
Scatterplot and linear correlation between medial proximal tibial angle (MPTA) and proximal fibular curvature (PFC).

Multivariate linear regression analysis indicated that PFC was negatively correlated with MPTA, with the regression equation as MPTA = 7.827+ 0.201 PFC (Table 5, Fig. 5). No linear correlation was revealed between MPTA and DFC, nor between MPTA and tibial curvature.

**Table 5:** Correlations between tibial and fibular curvatures and MPTA by multivariate linear regression analysis

Constant	Partial regression coefficient	Standardized coefficients β	*t*‐Value	*P*‐Value	95% CI		
	7.827	‐	0.384	0.701	−32.209	‐	47.863
PTC	0.020	0.014	0.326	0.745	−0.100	‐	0.140
DTC	0.119	0.077	1.820	0.069	−0.009	‐	0.246
PFC	0.201	0.139	3.296	0.001	0.081	‐	0.321
DFC	0.099	0.071	1.706	0.089	−0.015	‐	0.214

PTC, proximal tibial curvature; DTC, distal tibial curvature; PFC, proximal fibular curvature; DFC, distal fibular curvature; CI, confidence interval.

## Discussion

The S‐shaped fibula is anchored to the tibia *via* the proximal and distal tibiofibular joints and the interosseous membrane, and functions primarily for dissipation of loads applied at the ankle and stabilizing the ankle mortise, dissipation of lateral tibial bending moments, and transmission of axial loads in weight‐bearing^16^. Goh *et al.* reported that the load transmission through the fibula varied with the ankle position, and maximum loads occurred at full dorsiflexion and eversion^13^. Foot pronation, containing rearfoot eversion, is a compensation for varum malalignment of the knee to enable the foot to be plantigrade when weight‐bearing^17^. In physiologic loading conditions, the fibula sustained posterolateral bending force, and motions of the tibiofibular joints responding to various ankle positions and knee movements were mainly horizontal translation and rotation^14^, ^16^, ^18^, ^19^, ^20^. Therefore, we supposed that when the axial load distributed to the fibula increased in medial compartment knee OA patients due to the compensatory foot eversion, without vertical displacement of the proximal tibiofibular joint to dissipate the increased load, resultant bending of the fibula would occur.

### *Medial Tibial Settlement and Proximal Curve of the Fibula Increased in Knee Osteoarthritis Patients*

The results of this study showed that the MPTA, PTC, and PFC were significantly decreased in the knee OA group (K–L grade II–IV) compared to the non‐knee OA group (K–L grade I), while the K–L classification shifted to higher grades with age in all research objects (*χ* ^2^ = 182.169, *P* < 0.01). These results indicated that as the knee OA progressed with age, the medial joint space became narrower, and the non‐uniform settlement of the tibial plateau also progressed, resulting in increased tibia vara and varus malalignment of the knee. At mean time, the proximal fibular curve increased in patients with medial compartment knee OA. Fibular bowing was reported due to tibial shortening in an isolated fracture of the tibia back in 1986^21^. However, no similar finding was reported in the literature relating to knee OA. This anatomical change of the fibula may be an adaptation to the varus malalignment of the knee.

### *Medial Tibial Settlement and Fibular Curves Increased with Age*

Multivariate linear regression analysis showed that the MPTA, PFC, and DFC were all negatively correlated with age (*t* = −4.260, *P* = 0.000; *t* = −2.913, *P* = 0.004; *t* = −3.142, *P* = 0.002, respectively). The simultaneous progression of tibia vara and fibular bending with age was an interesting finding of the current study, indicating that both the non‐uniform settlement of the tibial plateau and bending of the fibula were in continuous progressing when the knee OA patients were aging.

### *Proximal Curve of the Fibula Increased with Medial Tibial Settlement*

Multivariate linear regression analysis showed that the PFC was positively correlated with the MPTA (*t* = 2.687, *P* = 0.007), indicating that the proximal curve of the fibula increased along with the non‐uniform settlement of the tibial plateau. This finding was consistent with our hypothesis that the anatomical change of the fibula was an adaptation to the varus malalignment in patients with medial compartment knee OA.

The findings of this current study further confirmed that tracecular collapse due to osteoporosis in aging individuals and lateral support provided by the fibula interacted, resulting in exacerbation of the varus deformity of the tibia, and consequent clinical and radiological progression of medial compartment knee OA.

### *Kinematic Effect of Fibula and Proximal Partial Fibulectomy in Treating Medial Knee Osteoarthritis*

The medial compartment knee load has been shown to be significantly correlated with the knee adduction moment (KAM)^22^. Solomonow‐Avnon *et al.* reported that the ground reaction force (GRF) and the knee lever arm (KLA) (i.e. the perpendicular distance from the knee joint center to the GRF), were significant predictors of KAM, and that KLA changed significantly with medial–lateral shift of the foot center of pressure (COP), while GRF did not. They concluded that a lateral shift in COP result in shortening of the KLA, and, consequently, reduced the KAM^23^. In patients with varus knee, the compensatory foot pronation reduces KAM by shifting COP laterally, and, thus, alleviates the medial compartment mechanic loading^7^, ^24^, ^25^, ^26^. Al‐Bayati reported that patients with supinated foot posture revealed the highest total WOMAC score, as well as the highest pain and physical function subscores, which would be a consequence of increased mechanic loading and cartilage degeneration due to the increased KAM^25^.

Foot pronation may increase the lateral ankle stress while protecting the medial compartment of the knee. Clinical studies have revealed the association between foot/ankle problems and the knee OA^27^, ^28^, ^29^. A stereophotographic study showed that foot pronation resulted in a more laterally located and smaller talofibular contact, which forced the distal fibula more laterally and corresponded to the greatest cartilage deformation, therefore indicating the greatest load^30^. In addition, the degree of genu varum that can be compensated by foot pronation depends on the available range of motion of the ankle, subtalar, and midtarsal joints^31^.

The lateral wedge insole used for knee OA patients was reported to significantly increase the amount of ankle/subtalar joint complex, resulting in lateral shift of the COP and reduction of KAM^32^, ^33^, ^34^, ^35^. However, accentuation of rearfoot pronation in already pronated feet could potentially result in higher talofibular contact pressure, causing ankle symptoms.

Compared to the lateral wedge insole, the proximal partial fibulectomy could allow more ankle eversion without increasing fibular loading, because of higher mobility of the distal fibula. Distal migration of the fibula head after proximal partial fibulectomy was revealed in our previous study^6^. Besides this change, talar tilt and proximal migration of the lateral malleolus, as well as significant reduction of load through the distal fibular remnant have been reported after partial fibulectomy^13^, ^36^, ^37^. Through receptive foot pronation, proximal partial fibulectomy significantly decreased the KAM and increased the hip–knee–ankle angle, thus alleviating the medial compartment loading to achieve pain and functional score improvement in knee OA patients^6^, ^7^, ^8^, ^9^, ^10^.

In patients with knee OA, significantly lower peak muscle activity of the biceps femoris and higher peak muscle activity of the peroneus longus were found compared to healthy controls. After proximal partial fibulectomy, muscle activity of the biceps femoris caput longum increased and remained high at 6 months, while that of the peroneus longus decreased 1 day after surgery and then recovered gradually^7^. The peroneus longus functions to pull the proximal half of the fibula laterally to promote its bending, and pull the fibular head distally after partial fibulectomy, resulting in tension of the biceps femoris and the lateral collateral ligament of the knee joint, which could facilitate the lateral shift of knee joint load.

In conclusion, this study showed that the proximal curve of the fibula increased in patients with medial compartment knee OA, and this change was positively correlated with age and settlement of the medial tibial plateau. This anatomical adaptation of the fibula was associated with greater fibular axial load and the pulling from the peroneus longus. The proximal partial fibulectomy procedure effected a receptive foot pronation to reduce KAM and rebalance the biceps‐proximal fibula–peroneus longus complex, consequently achieving medial compartment unloading.

This study was a preliminary investigation focusing on the anatomy of the fibula based on its coronal projection by standing AP full‐leg radiography. The results may potentially underestimate the change of fibular curves. Measurement errors may be caused by manually drawing lines on the X‐ray images. In addition, the sample size was insufficient for stratified analysis by K–L grades of the knee OA.

The fibula integrity and motion affect the knee kinematics *via* changes at the ankle and foot. As such, we suggest including ankle/foot screening for knee OA patients to appropriately identify those who are most likely to benefit from the proximal partial fibulectomy procedure. Prospective comparisons of skeletomuscular changes of the fibula and foot/ankle between preoperative and postoperative periods using three‐dimensional models need to be carried out to validate the effective mechanism and to define surgical indications of proximal partial fibulectomy in treating medial compartment knee OA.
